# Physicians’ Mutual Benefit Association of Texas; Statement of the Secretary; Operations and Disbursements

**Published:** 1888-01

**Authors:** 


PHYSICIANS' MUTUAL BENEFIT ASSOCIATION OF TEXAS. STATEMENT. The Association was organized and chartered April, 1884. The total number of members who have joined to date is 211. Of these 47 have forfeited their membership for non-payment of assessments or annual dues, (nine having failed to pay assessment No. 4 for Mrs. Kilpatrick,) and five have died, leaving an active membership of 159; and two having joined since the date of Dr. Starley's death, 157 are liable to assessment No. 5, which has been issued. The annual dues of $1.00 for 1888 has been paid by all but twenty-members, and as the time for payment has passed there is no way of knowing how many of these will fail to be re-instated. and assessment No. 5 will be affected proportionately, Second notices have been sent to all in arrears, and a list will be published in next issue of those who pay the dues for 1888.

The following table shows the net results of the four assessments issued prior to January, 1888. It will be seen that four hundred and twenty-seven dollars and seventy cents ($427.70) has been paid to the families of the four physians who died during the four years, an average of $106.92 to each family:

| | |
|---|---|
| Certificate No. 26, Dr. W. H. Park; died Nov. 4, 1885; 61 members assessed; paid Mrs. Park............$ | 55 00 |
| Certificate No. 89, Dr. Sayers; died July, 1886; 121 members assessed; paid Mrs. Sayers.................. | 109 00 |
| Certificate No. 134, Dr. Welch; died Sep. 1886; 132 members assessed; paid Mrs. Welch............. | 120 00 |
| Certificate No. 46, Dr. Kilpatrick; died Sep. 1887; 157 members assessed; paid Mrs. Kilpatrick........ | 143 70 |
| Total amount of benefits paid ...................... $ | 427 70 |

With the exception of Dr. Kilpatrick none of the members had paid in more than the initiation fee.

Apropos: In last issue we said that Dr. W. T. Burt would receive application for membership in the P. M. B. A.; beg pardon; we *meant* to say Dr. W. P. Burts, of Fort Worth.

